# Transmembrane protein 176B regulates amino acid metabolism through the PI3K-Akt-mTOR signaling pathway and promotes gastric cancer progression

**DOI:** 10.1186/s12935-024-03279-4

**Published:** 2024-03-04

**Authors:** Jing Li, ZiQing Fang, Emre Dal, Hao Zhang, KeXun Yu, MengDi Ma, MingLiang Wang, Ruochuan Sun, MingDian Lu, HuiZhen Wang, YongXiang Li

**Affiliations:** 1 Department of General Surgery, The First Affiliated Hospital of Anhui Medical University, Hefei, 230022 China; 2 Department of Gastrointestinal Surgery, The First Affiliated Hospital of Anhui Medical University, Hefei, 230022 China; 3 University of Utah, Salt Lake City, UT 84102 USA

**Keywords:** Asparagine synthetase, Gastric cancer, Mammalian target of rapamycin, Phosphoinositide 3-kinase, Protein kinase B, Transmembrane protein 176B

## Abstract

**Background:**

The present study aimed to investigate the expression level, biological function, and underlying mechanism of transmembrane protein 176B (TMEM176B) in gastric cancer (GC).

**Methods:**

TMEM176B expression was detected by quantitative real-time polymerase chain reaction (qRT-PCR) and western blotting (WB). The function of TMEM176B was determined by various in vitro assays including colony formation, 5-ethynyl-2ʹ-deoxyuridine (EdU), Transwell, and flow cytometry. Bioinformatics techniques were then used to elucidate the signaling pathways associated with TMEM176B activity. Tumor formation experiments were conducted on nude mice for in vivo validation of the preceding findings. TMEM176B expression was cross-referenced to clinicopathological parameters and survival outcomes.

**Results:**

It was observed that TMEM176B was overexpressed in GC cells and tissues. Targeted TMEM176B abrogation inhibited colony formation, proliferation, migration, and invasion but promoted apoptosis in GC cell lines while TMEM176B overexpression had the opposite effects. Subsequent experimental validation disclosed an association between TMEM176B and the phosphatidylinositol 3-carboxykinase (PI3K)-protein kinase B (Akt)-mammalian target of rapamycin (mTOR) signaling axis. Moreover, TMEM176B affects GC cancer progression by regulating asparagine synthetase (ASNS). The in vivo assays confirmed that TMEM176B is oncogenic and the clinical data revealed a connection between TMEM176B expression and the clinicopathological determinants of GC.

**Conclusion:**

The foregoing results suggest that TMEM176B significantly promotes the development of gastric cancer and is an independent prognostic factor of it.

**Supplementary Information:**

The online version contains supplementary material available at 10.1186/s12935-024-03279-4.

## Introduction

Gastric carcinoma (GC) is a major malignancy with high global incidence and mortality rates [1, 2]. China in particular and East Asia in general account for ≤ 44% of all new GC diagnoses worldwide [2]. When GC is detected early, its 5-year survival rate is > 90% [3]. East Asia, particularly China, is disproportionately burdened with this neoplastic disease, contributing nearly 44% of all novel diagnoses on a global scale [4]. Chemotherapy and targeted treatment modalities have only limited efficacy in advanced GC cases [5, 6]. Hence, innovative timely prophylactic and therapeutic approaches and interventions for GC are urgently required.

Members of the heterogeneous transmembrane protein (TMEMs) family are embedded within the lipid bilayers [7, 8] of cell and organellar membranes [9]. They promote angiogenesis [10], regulate endoplasmic reticulum (ER) stress [11, 12], maintain mitochondrial function [13, 14], facilitate protein glycosylation [15], control epidermal keratinization [16], and modulate smooth muscle contraction [17].

The multifunctional TMEM176B is a key member of the TMEM family. It was found to be upregulated in a rat model of allograft tolerance and designated as *tolerance-related and induced transcript* (TORID). Other functions of TMEM176B have since been identified [18]. TMEM176B forms a co-polymer with TMEM176A, retards dendritic cell (DC) maturation, and may regulate DCs overall [19]. TMEMs are also associated with antigen delivery in DCs and the modulation of DC-mediated immune responses [20, 21]. TMEM176B bolsters antitumor immunity and synergistically increases immune checkpoint blockade efficacy by activating inflammasomes [22]. TMEM176B promotes triple-negative breast cancer progression by regulating the Akt/mTOR signaling pathway [23]. TMEM176B expression in melanoma indicates a favorable prognosis. Thus, TMEM176B could serve as both a diagnostic and a prognostic marker for certain malignancies [24]. It may also be a melanoma immunotherapy target as it regulates CD8 + T cells. However, the mechanisms of TMEM176B in the onset and progression of GC remain to be clarified.

Phosphatidylinositol 3-kinases (PI3Ks) are crucial coordinators of intracellular signalling in response to the extracellular stimulators. Hyperactivation of PI3K signalling cascades is one among the most ordinary events in human cancers [25]. As an intracellular protein complex, rapamycin complex 1mTORC1 consists of five components, namely, mTOR, RAPTOR, DEPTOR, mLST8 and PRAS40 [26]. mTORC1 can integrate signals from growth factors and nutrients to control biosynthesis, including protein, lipid and nucleic acid synthesis [27]. Phosphatidylinositol 3-kinases (PI3Ks) are crucial coordinators of intracellular signalling in response to the extracellular stimulators. Hyperactivation of PI3K signalling cascades is one among the most ordinary events in human cancers [28]. Asparagine synthetase (ASNS) is a key enzyme in amino acid (AA) metabolism [29, 30]. It generates asparagine from glutamine-derived nitrogen and aspartate [31] and is implicated in lung, ovarian, and gastric cancer progression [32–34].

The present study aimed to elucidate the molecular mechanisms of TMEM176B and identify novel therapeutic targets and optimize treatment strategies for gastric cancer.

## Materials and methods

### Bioinformatics analysis

GC-related transcriptomic data were procured from The Cancer Genome Atlas (TCGA) database (https://tcga-data.nci.nih.gov/tcga/) and subjected to bioinformatics analysis with R v. 4.1.2 (https://cran.r-project.org/bin/windows/base/old/4.1.2/). Additional bioinformatics analyses were conducted on TCGA-stomach adenocarcinoma (STAD) data (https://linkedomics.org/data_download/TCGA-STAD/) to establish the molecular mechanism of TMEM176B in GC. The samples were divided into the high- and low TMEM176B expression groups. Differentially expressed genes (DEGs) between groups were detected using the selection criteria |log fold change (FC)|> 1 and adjusted P-value and subjected to downstream gene ontology (GO) enrichment analysis of cellular components, molecular functions, and biological processes as well as Kyoto Encyclopedia of Genes and Genomes (KEGG) enrichment analysis of biological. The “clusterProfiler” package (https://bioconductor.org/packages/release/bioc/html/clusterProfiler.html) in R was used to perform the GO and KEGG analyses and the “ggplot2” package (https://rdrr.io/r/utils/install.packages.html) in R was used to visualize the output.

### Cell culture

The gastric epithelial cell lines GES-1, MGC803, HGC27, AGS, and SGC7901 were sourced from GeneChem (Shanghai, China) and cultivated in RPMI-1640 medium (Corning Life Sciences, Corning, NY, USA) supplemented with 10% (v/v) fetal bovine serum (FBS; Clark Bioscience, Richmond, VA, USA) and 1% (w/v) penicillin plus 1% (w/v) streptomycin (HyClone Laboratories, Logan, UT, USA). All cell lines were maintained at 37 ℃ under a humidified 5% CO_2_ atmosphere.

### Quantitative real-time polymerase chain reaction (qRT‒PCR)

Total RNA was extracted from GC cells and tissues with TRIzol Reagent (Invitrogen, Carlsbad, CA, USA) per the manufacturer’s instructions [35]. The RNA was then reverse-transcribed to cDNA and the latter was subjected to qRT-PCR according to standard procedures. The primer sequences used in this study are shown in Additional file 1: Table S1.

### Western blotting

The proteins from the GC cells and tissues were extracted with M-per protein lysis buffer (Thermo Fisher Scientific, Waltham, MA, USA) supplemented with protease and phosphatase inhibitors (BBI Life Sciences Corporation, Shanghai, China) and subjected to western blotting [36] with the primary antibodies anti-GAPDH (No 7074 T; Cell Signaling Technology (CST), Danvers, MA, USA), anti-TMEM176B (No. PHC0758; Abmart, Wuhan, China), anti-ASNS (No. R22614; ZenBio, Chengdu, China), anti-AKT (No. R23411; ZenBio), anti-p-AKT (No. R381555; ZenBio), anti-PI3K (No. R381092; ZenBio), anti-p-PI3K (No. 310164; ZenBio), and anti-PI3K (No. R381092; ZenBio). The mTOR Substrates Antibody Sampler Kit (No. 9862 T) was sourced from CST.

### Patient samples and follow-up

One hundred and seven formalin-fixed, paraffin-embedded GC and 22 adjunct normal tissues were curated from The First Affiliated Hospital of Anhui Medical University between October 2012 and December 2013. None of the patients with GC had undergone preoperative chemotherapy or radiotherapy, and all of them were followed up for 8–71 mo. The clinicopathological data and staging based on the American Joint Committee on Cancer (AJCC) v. 8 system are displayed in Table 1. Ethical approval of the research protocol was obtained from the Ethics Association of Anhui Medical University, and all eligible trial participants provided written informed consent.

**Table 1:** Correlation of TMEM176B expression with clinicopathologic parameters in GC patients

Parameters	Cases	TMEM176B expression		**χ**^2^	*P*-Value
		Low	High		
Gender				0.045	0.833
Male	71	39	32		
Female	36	19	17		
Age(years)				0.448	0.503
< 61	43	25	18		
≥ 61	64	33	31		
Tumor location				0.045	0.833
Upper	36	19	17		
Middle + lower	71	39	32		
Tumor size(cm)				9.696	0.002^a^
< 6	48	34	14		
≥ 6	59	24	35		
Depth of invasion				5.313	0.021^a^
T1 + T2	29	21	8		
T3 + T4	78	37	41		
Lymph node metastasis				4.584	0.032^a^
Absent	23	17	6		
Present	84	41	43		
Differentiation				7.313	0.007^a^
Well + moderate	41	29	12		
Poor	66	29	37		
TNM stage				5.388	0.020^a^
I + II	34	24	10		
III + IV	73	34	39		

^a^Statistically significant (*p* < 0.05)

### Immunohistochemical (IHC) staining

Tissue microarrays (TMAs) were subjected to IHC staining per established protocols [37] to quantify TMEM176B expression. For this purpose, anti-TMEM176B (No. PHC0758; 1:1,000; Abmart) was used. Staining intensity ranged from 0 (none) to 3 (strong). The staining area ranged from 0 (none) to 4 (76–100%). Assessments were made independently by two expert pathologists. The staining score was the product of staining intensity and staining area. Values ≥ 5 and those in the range of 0–4 indicated high and low TMEM176B expression, respectively.

### Cell lentivirus infection

Three small hairpin RNAs (shRNAs) and a TMEM176B overexpression lentivirus were acquired from GeneChem. GC cells were seeded in a 12-well plate and transfected with the lentivirus at Multiplicity of Infection (MOI) = 10 per the manufacturer’s guidelines. Successful transfection was confirmed by using a medium supplemented with 2 mg/mL puromycin, and the transfected cells were maintained in a medium supplemented with 1 mg/mL puromycin. The shRNA sequences used were as follows:

sh#1: 5ʹ-GUAGGUCUUCGAAACUUGUTT-3ʹ

sh#2: 5ʹ-GCAGGCUUUGCUACAGCUATT-3ʹ.

### Colony formation assay

GC cells were inoculated into a six-well plate at a density of 800/well. The medium was replenished every 3rd day. Starting at day 7, the cultures were inspected for clonal growth and the culture was terminated as soon as clones could be detected with the unaided eye. The cells were then fixed with 4% (v/v) paraformaldehyde (PFA) and stained with 0.1% (w/v) crystal violet. The clones in each well were counted and the stained cells were air-dried and imaged.

### EdU assay

The present assay was conducted using an EdU Kit (No. C0078S; Beyotime Biotechnology, Shanghai, China). Sterile slides were set in 12-well plates, seeded with cells, and incubated overnight to the optimal density. The cells were then exposed to 2X EdU solution, rinsed with phosphate-buffered saline (PBS) plus 3% (v/v) bovine serum albumin (BSA), permeabilized with 0.3% (v/v) Triton X-100, and rinsed. A Click reaction solution was added to the 12-well plate and the cells were incubated in it for 30 min. The nuclei were then stained with Hoechst 33,342 for 10 min. The cells were subjected to an anti-quenching agent and examined under a microscope (Leica Microsystems, Wetzlar, Germany).

### Transwell assay

Transwell assay was performed as described previously [38]. For invasion assay, GC cells in log phase were incubated into the Transwell chamber (Corning Life Sciences, Corning, NY, USA) at the optimal density in serum-free medium for 24 h. Matrigel (BD Biosciences, Shanghai, China) was diluted and uniformly applied to the upper Transwell chamber and the treated cells were incubated at 37 ℃ for 5 h. A cell suspension containing 1 × 10^8^ cells and serum-rich medium were introduced into the upper- and lower Transwell chambers, respectively. After a predetermined incubation period, the Transwell chambers were treated with 4% (v/v) PFA, stained with 0.1% (w/v) crystal violet, and examined and imaged under a microscope (Leica Microsystems). The migration assay does not use Matrigel and the rest of the steps are the same as the invasion assay. The incubation time was 28 h for HGC27 cells, 24 h for SGC7901 cells, and 18 h for AGS cells.

### Flow cytometry

Flow cytometry was performed as described previously [39]. This assay was performed with an Annexin V-FITC/PI Apoptosis Kit (Yeason Biotechnology, Shanghai, China). 2 × 10^5^ HGC27, SGC7901, or AGS GC cells were cultured overnight in 12-well plates, respectively. GC cell suspensions were washed with PBS, resuspended at a 1:1 (v/v) ratio in binding buffer, stained with Annexin V-FITC (fluoroisothiocyanate) and PI (propidium iodide) reagents, and incubated in the dark for 15 min. Apoptosis was detected by CytoFLEX flow cytometry (Beckman Coulter, Brea, CA, USA).

### Xenograft mouse model

BALB/C nude mice were procured from GemPharmatech, Jiangsu, China and acclimated under specific pathogen-free (SPF) conditions for 4 weeks. Each mouse was subcutaneously injected in the left axilla with 5 × 10^6^ enzymatically digested GC cells in PBS suspension. Throughout the experiment, the mice had ad libitum food and water access and their body weight and tumor dimensions were recorded every 3 day. Mice exhibiting ≥ 20% weight loss and/or tumor diameter > 1.5 cm were humanely euthanized.

### Statistical analysis

All data were subjected to Student’s *t*-test or one-way analysis of variance (ANOVA) and otherwise processed with SPSS v. 22.0 (SPSS, Inc., Chicago, IL, USA), GraphPad Prism 7.0 (GraphPad Software, La Jolla, CA, USA), and R v. 4.1.2. p < 0.05, p < 0.01, and p < 0.001 indicated statistical significance.

## Results

### TMEM176B was significantly upregulated in GC cells and tissues

**Fig. 1:**
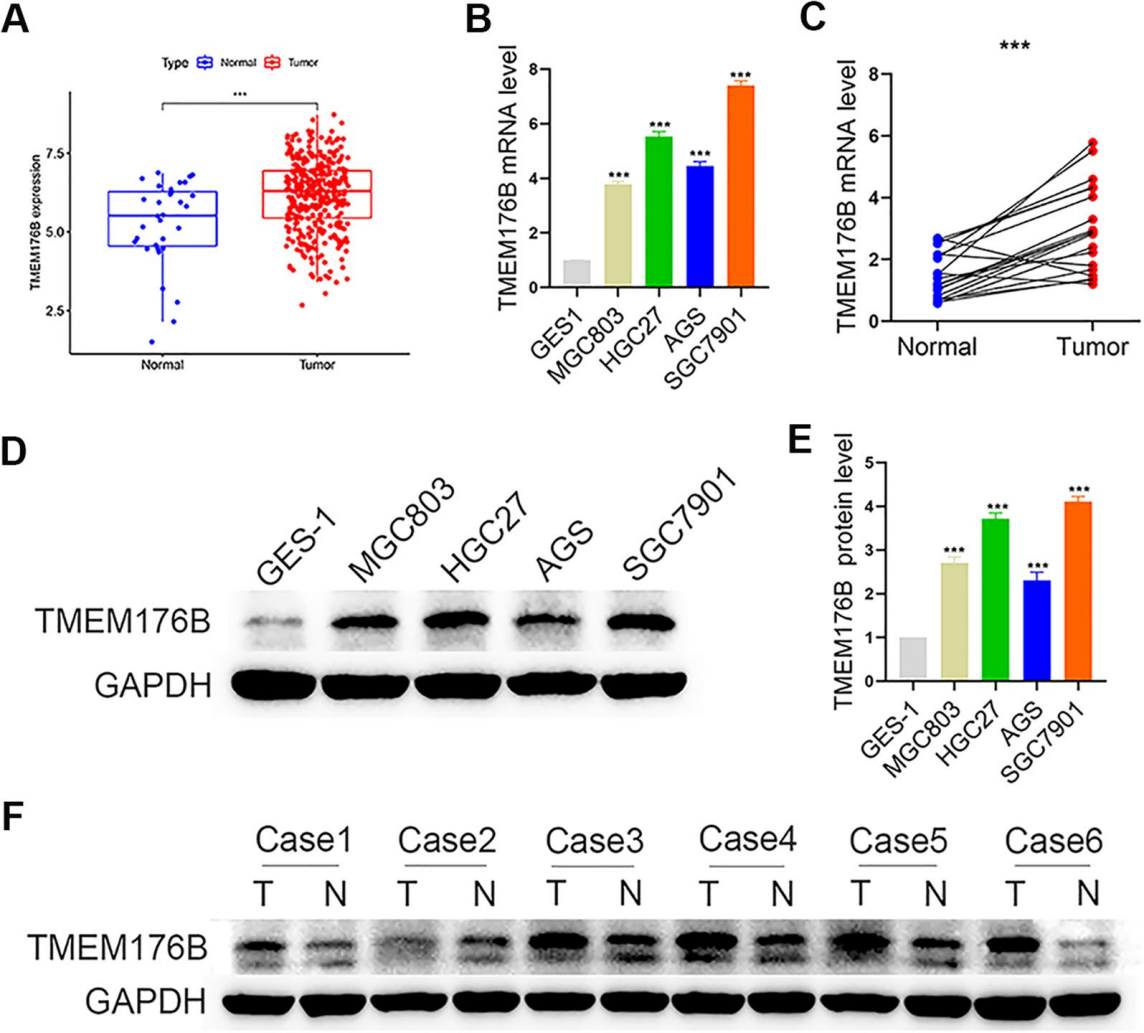
TMEM176B was significantly upregulated in gastric cancer (GC). **A** TMEM176B expression in GC was determined from The Cancer Genome Atlas (TCGA) database. **B**, **C** TMEM176B RNA expression in GC cell lines and tissues was detected by qRT-PCR. **D**, **E** Western blot (WB) of TMEM176B protein expression in GES-1 and GC cell lines. **F** WB of TMEM176B protein expression in GC and adjacent non-neoplastic tissues. *T* tumor tissue, *N* normal tissue. ^*^p < 0.05, ^**^p < 0.01, ^***^p < 0.001

We conducted a bioinformatics analysis of data from TCGA database and focused specifically on TMEM176B expression in GC. As TMEM176B was upregulated in GC, it might participate in the pathogenesis of this disease (Fig. 1A).

We then used PCR to compare TMEM176B mRNA expression in the MGC803, HGC27, AGS, and SGC7901 GC cell lines against that in the normal gastric epithelial cell line GES1. The bioinformatics and PCR analyses showed that TMEM176B mRNA expression was higher in the gastric cancer cell lines than in GES1 (Fig. 1B).

The observation that the TMEM176B mRNA levels were significantly higher in most GC tissues than in their adjacent normal tissues validated our initial hypothesis (Fig. 1C).

We then applied western blotting (WB) to verify TMEM176B protein expression in the GC cell lines and tissue samples. Similarly, the TMEM176B protein levels were considerably higher in the GC cell lines and tissues than in their normal counterparts (Fig. 1D-F).

Taken together, the foregoing findings suggested that TMEM176B is upregulated in GC cells and tissues and justified the subsequent investigations into the mechanisms of TMEM176B in GC onset and progression.

### TMEM176B knockdown suppressed the proliferation, migration, and invasion and enhanced the apoptosis of GC cells

**Fig. 2:**
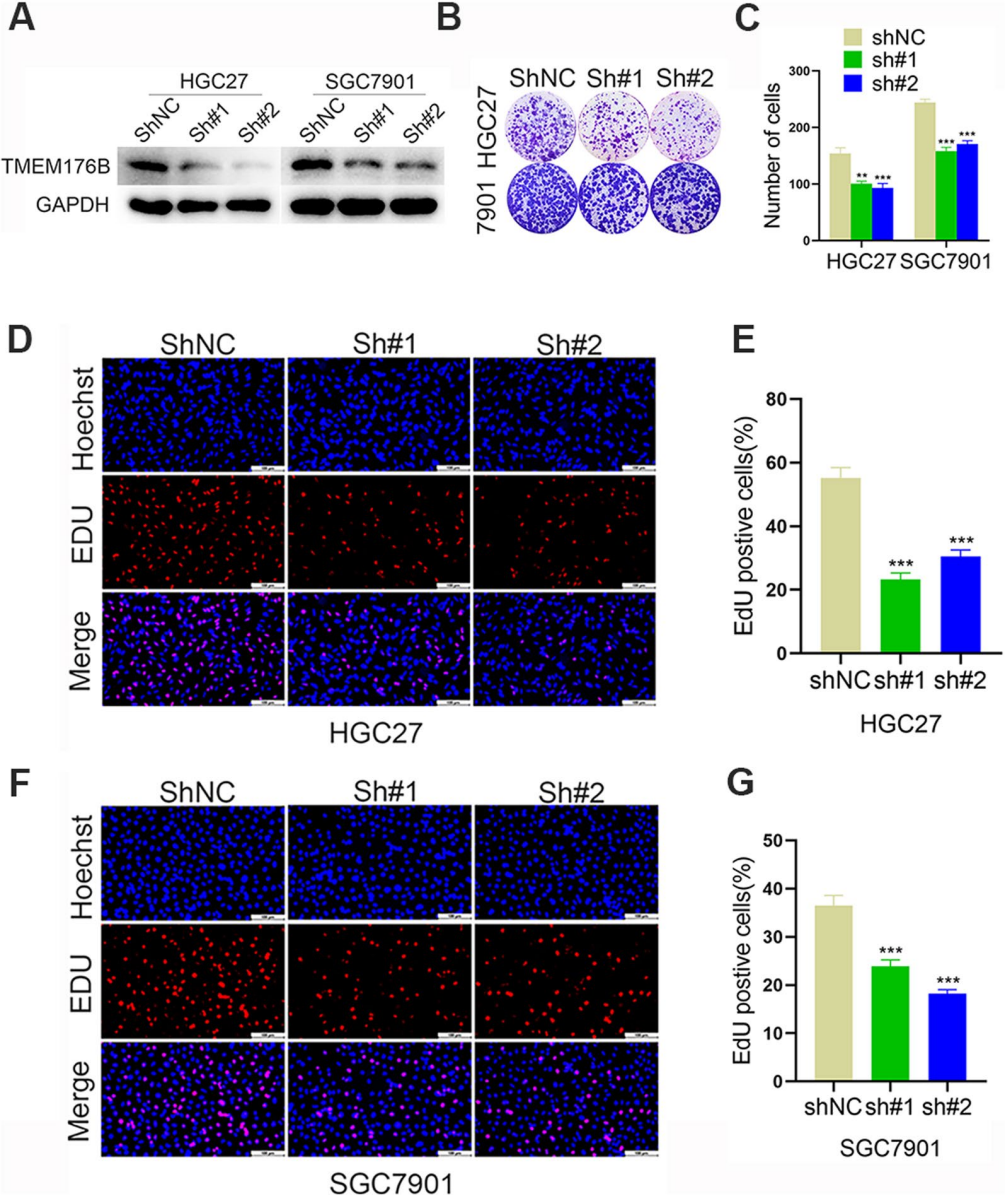
TMEM176B downregulation suppressed GC clonal formation and proliferation. **A** WB determined TMEM176B knockdown efficiency in HGC27 and SGC7901 cell lines. **B**, **C** Impact of TMEM176B silencing on the clonogenic potential of HGC27 and SGC7901 cell lines. **D**, **E** Impact of TMEM176B silencing on HGC27 cell line proliferation was assessed by EdU assay. **F**, **G** Impact of TMEM176B silencing on SGC7901 cell line proliferation was assessed by EdU assay. ^*^p < 0.05, ^**^p < 0.01, ^***^p < 0.001

We then investigated the functions of TMEM176B in GC by downregulating it in the HGC27 and SGC7901 cell lines (Fig. 2A).

We used colony formation and EdU assays to evaluate the impact of TMEM176B knockdown on GC cell proliferation and found that the proliferation ability of gastric cancer cells was significantly decreased. Hence, TMEM176B is implicated in GC cell proliferation (Fig. 2B–G).

**Fig. 3:**
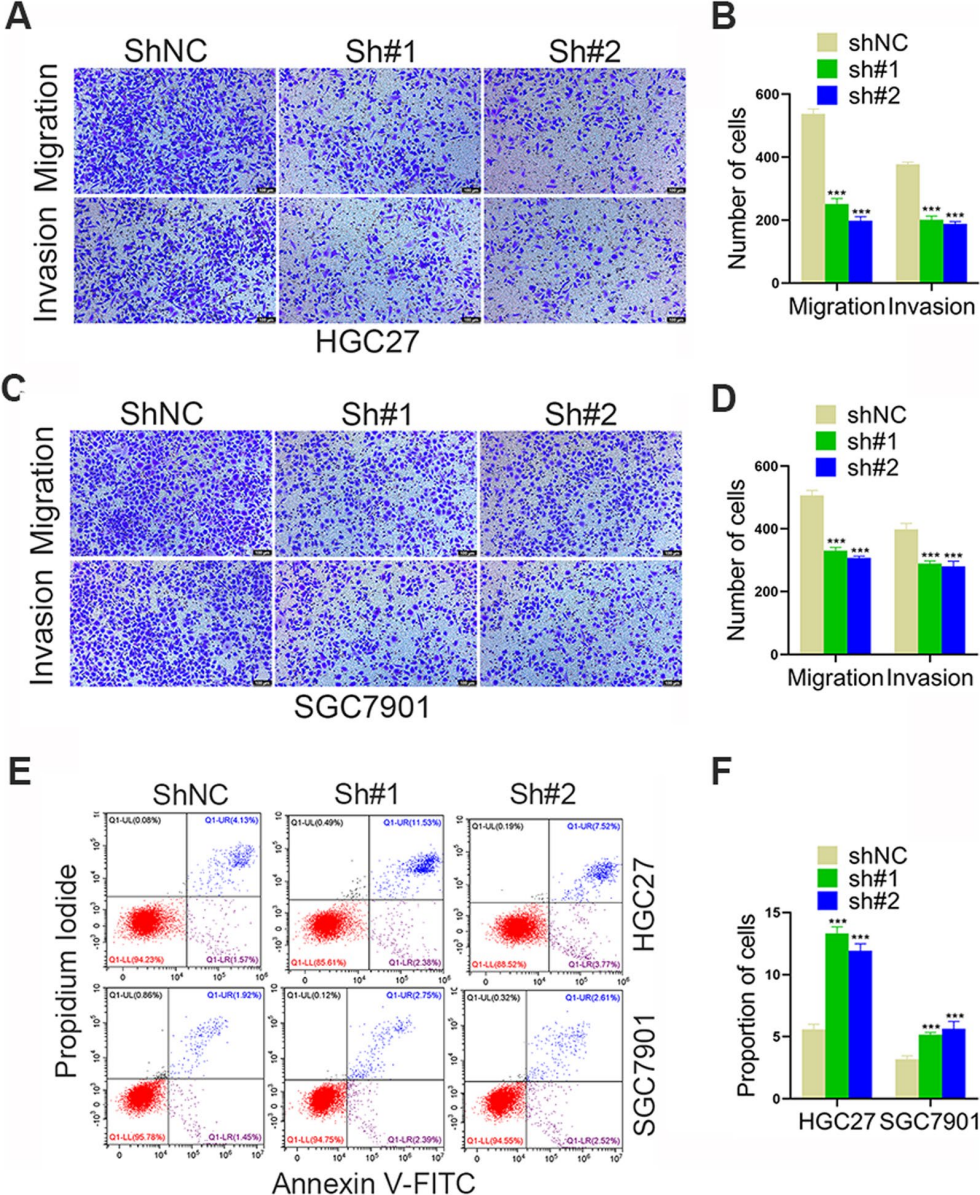
TMEM176B downregulation decreased proliferation, migration, and invasion and increased apoptosis in GC cell lines. **A**, **B** Impact of TMEM176B silencing on HGC27 cell line migration and invasion was assessed by Transwell assay. **C**, **D** Impact of TMEM176B silencing on SGC7901 cell line migration and invasion was assessed by Transwell assay. **E**, **F** Impact of TMEM176B silencing on HGC27 and SGC7901 cell line apoptosis. ^*^p < 0.05, ^**^p < 0.01, ^***^p < 0.001

We then used a Transwell assay to assess the effect of TMEM176B silencing on GC cell migration/invasion and found that migration and invasion ability decreased significantly (Fig. 3A–D).We then used flow cytometry to investigate whether TMEM176B knockdown affects GC cell apoptosis and found that the proliferation ability of gastric cancer cells was significantly decreased (Fig. 3E–F).

### TMEM176B overexpression promoted the proliferation, migration, and invasion and inhibited the apoptosis of GC cells

**Fig. 4:**
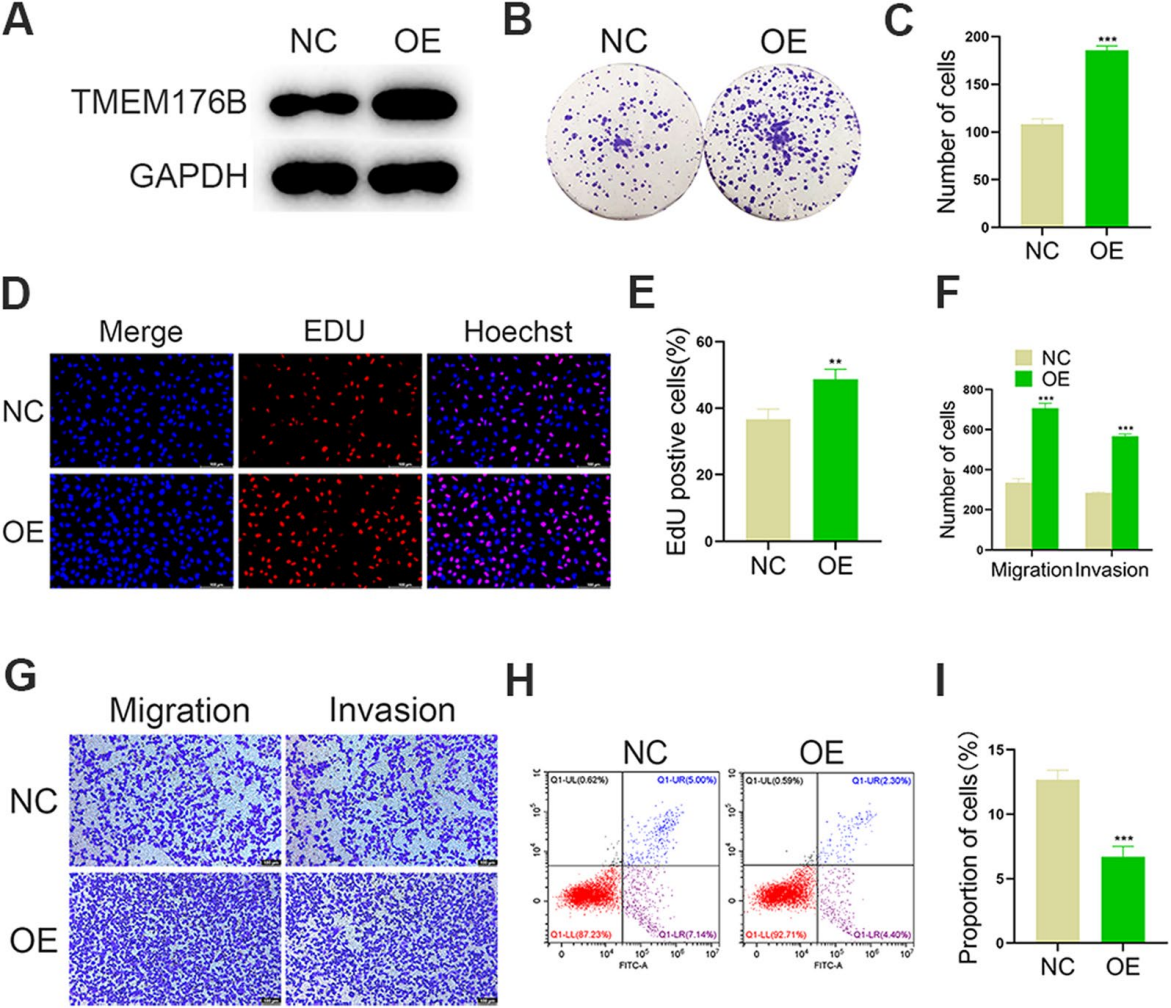
TMEM176B overexpression increases proliferation, migration, and invasion and decreases apoptosis in GC cell lines. **A** WB determined TMEM176B overexpression efficiency in AGS cell lines. **B**, **C** Impact of TMEM176B overexpression on the clonogenic potential of AGS cell lines. **D**, **E** Impact of TMEM176B overexpression on AGS cell line proliferation was assessed by EdU assay. **F**, **G** Impact of TMEM176B overexpression on AGS cell line migration and invasion was assessed by Transwell assay. **H**, **I** Impact of TMEM176B overexpression on AGS cell line apoptosis. *NC* normal control, *OE* overexpression; ^*^p < 0.05, ^**^p < 0.01, ^***^p < 0.001

We then overexpressed TMEM176B in AGS cell lines (Fig. 4A) to assess its impact on key processes in GC cells.

We used the results of the colony formation assay as baseline GC cell proliferation values. The GC cell colonies overexpressing TMEM176B were more numerous than the control (Fig. 4B, C). Thus, TMEM176B upregulation is associated with GC cell proliferation.

We validated the foregoing findings with an EdU assay and observed more EdU-positive proliferating cells in the TMEM176B overexpression treatment than in the control (Fig. 4D, E).

A Transwell assay revealed that TMEM176B overexpression significantly increased the number of GC cells compared to the control. Therefore, TMEM176B upregulation enhances GC cell migration and invasion (Fig. 4F-G).

We then assessed the effect of TMEM176B overexpression on GC cell apoptosis and noted that the The apoptosis ability of overexpression group was significantly decreased compared to the control. Hence, TMEM176B overexpression decreases programmed death in GC cells (Fig. 4H, I).

### *TMEM176B may promote GC progression by regulating *ASNS* metabolism through the PI3K-Akt-mTOR signaling pathway*

**Fig. 5:**
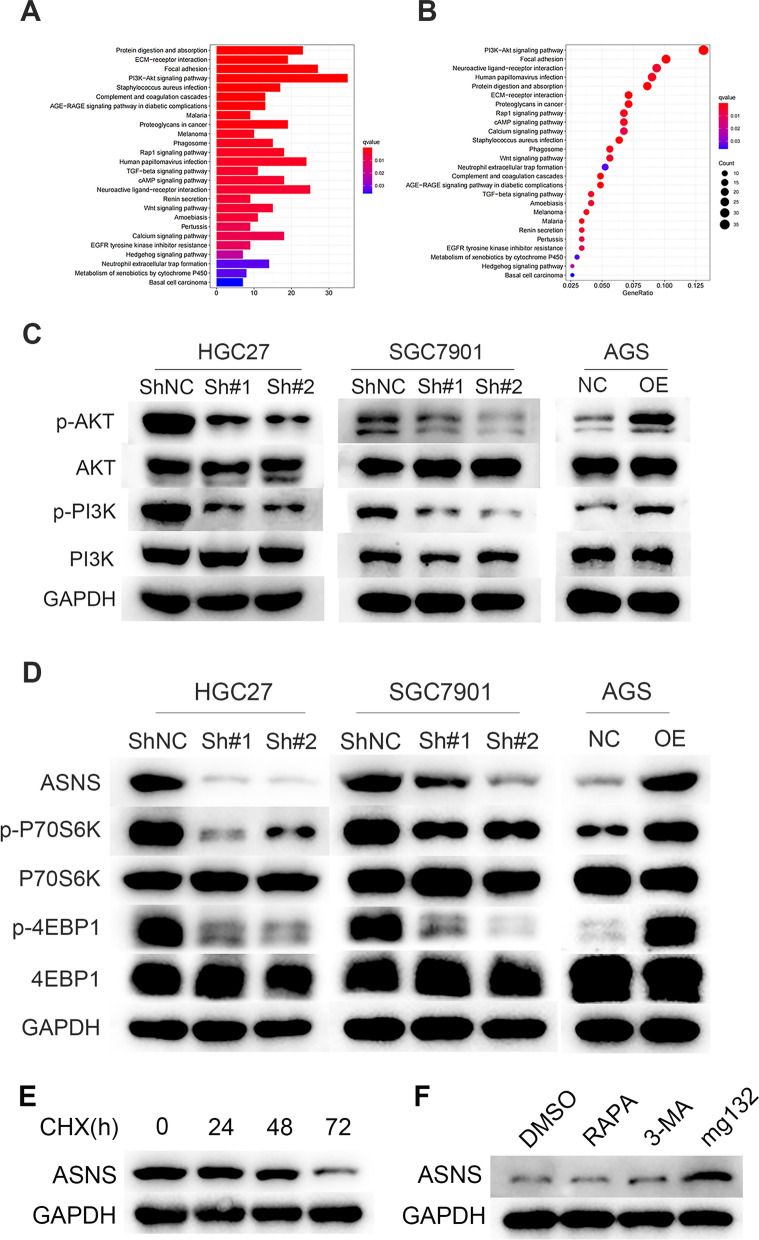
TMEM176B drives GC progression via the PI3K-Akt-mTOR signaling pathway. **A** Differentially expressed genes (DEGs) were subjected to KEGG pathway enrichment analysis. **B** Gene set enrichment analysis (GSEA) of DEGs. **C**, **D** WB determined the expression levels of ASNS proteins related to the PI3K-Akt-mTOR signaling pathway in GC cell lines after TMEM176B knockdown or overexpression. **E** HGC27 cell line with TMEM176B knockdown was treated with cycloheximide (CHX) to detect ASNS expression at various time points. **F** HGC27 cell line with TMEM176B knockdown was treated with dimethyl sulfoxide (DMSO), rapamycin, 3-methyladenine (3-MA), and MG132 to detect ASNS expression

We then conducted an enrichment analysis of various signaling pathways to elucidate the mechanisms by which TMEM176B affects GC progression. We detected a strong positive correlation between TMEM176B expression and the PI3K-Akt signaling pathway. The latter regulates cell survival, proliferation, and metabolism (Fig. 5A, B).

We used WB to probe the activity levels of the key proteins in the PI3K-Akt signaling pathway and found that TMEM176B knockdown markedly downregulated PI3K and Akt phosphorylation whereas TMEM176B overexpression had the opposite effects (Fig. 5C).

We also observed that TMEM176B silencing and overexpression decreased and increased, respectively, the phosphorylation states of ribosomal protein S6 kinase beta-1 (P70S6K) and eukaryotic initiation factor 4E-binding protein 1 (4EBP1) associated with mTOR, the downstream effector of the PI3K-Akt signaling cascade (Fig. 5D).

An earlier study reported that mTOR is closely connected to ASNS [40]. Thus, we explored whether there is an association between TMEM176B and ASNS expression in GC progression. In all GC cell lines, ASNS expression decreased and increased in response to TMEM176B knockdown and overexpression, respectively (Fig. 5D). Cycloheximide (CHX) addition indicated that TMEM176B inhibited ASNS degradation but did not promote its biosynthesis (Fig. 5E). We then added mTOR, autophagy, and ubiquitination inhibitors to the GC cell lines with TMEM176B silencing and observed that ASNS expression increased only in response to the addition of the proteasome inhibitor MG132. Therefore, TMEM176B induced ASNS by inhibiting its ubiquitination degradation (Fig. 5F).

The preceding findings suggest that TMEM176B promotes GC progression by regulating ASNS metabolism through the PI3K-Akt-mTOR signaling pathway.

### MTOR inhibition partially reversed malignant GC promotion by TMEM176B overexpression

**Fig. 6:**
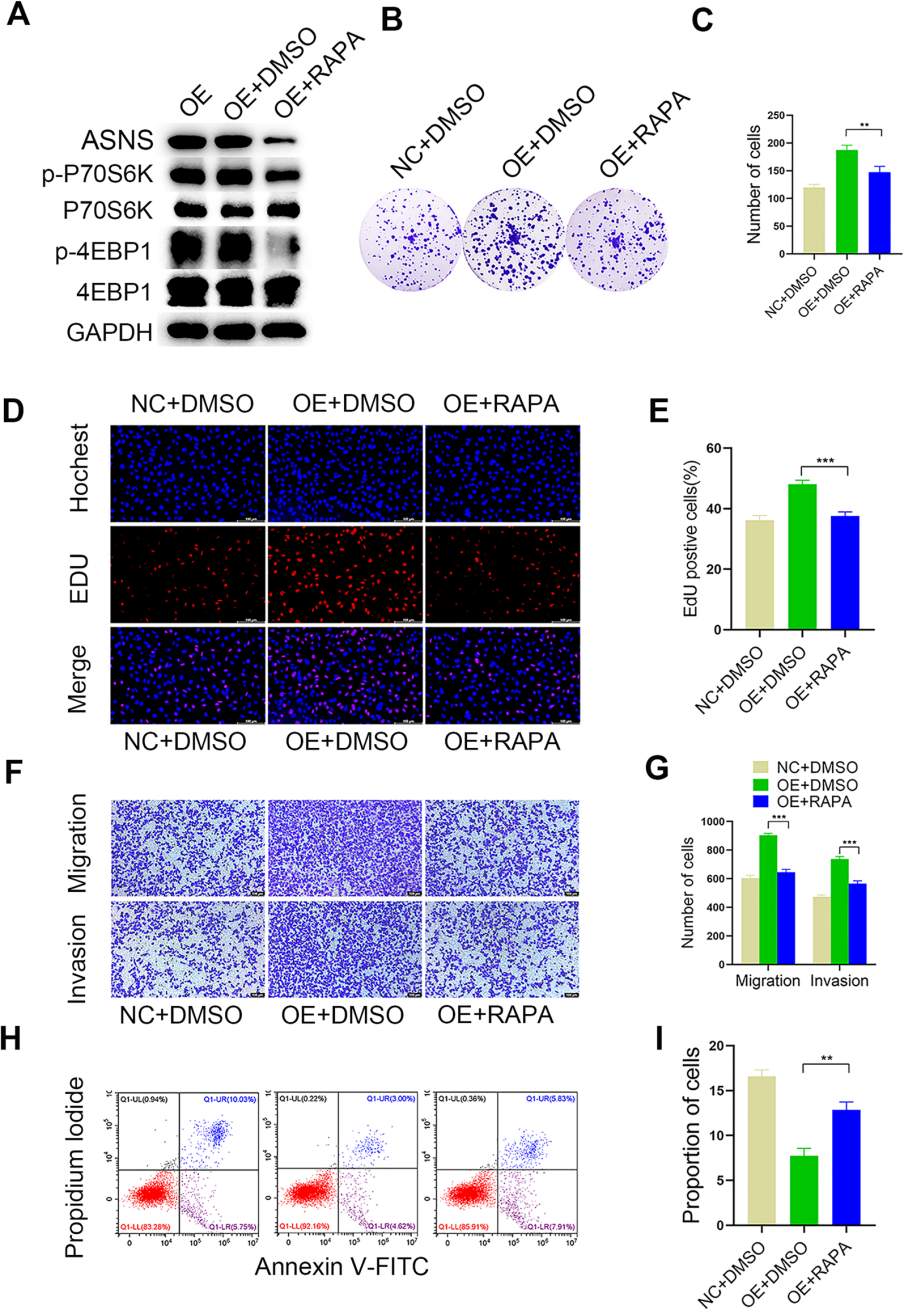
mTOR inhibitors partially reverse the malignant GC-promoting effect of TMEM176B overexpression. **A** The efficiency of mTOR inhibition by rapamycin was measured in AGS cell lines overexpressing TMEM176B. **B**, **C** Impact of rapamycin on the clonogenic potential of AGS cell lines overexpressing TMEM176B. **D**, **E** Impact of rapamycin on the proliferation of AGS cell lines overexpressing TMEM176B was assessed by EdU assay. **F**, **G** Impact of rapamycin on migration and invasion of AGS cell lines overexpressing TMEM176B was assessed by Transwell assay. **H**, **I** Impact of rapamycin on apoptosis in AGS cell lines overexpressing TMEM176B. *NC* normal control, *OE* overexpression, *DMSO* dimethyl sulfoxide, *RAPA* rapamycin;^*^p < 0.05, ^**^p < 0.01, ^***^p < 0.001

We treated AGS cells (characterized by TMEM176B overexpression) with the mTOR inhibitor rapamycin (50 nM) to clarify the interaction between TMEM176B and the PI3K-Akt-mTOR signaling cascade (Fig. 6A). We found that rapamycin addition significantly downregulated ASNS (Fig. 6A). We then used colony formation, EdU, Transwell, and flow cytometry assays to evaluate proliferation, DNA replication, migration-invasion, and apoptosis in the resultant GC cell phenotype.

Our data corroborated the hypothesis that rapamycin attenuates the stimulatory effects of TMEM176B overexpression on GC cell proliferation, migration, and invasion. In contrast, it counteracts the inhibitory effect of TMEM176B overexpression on GC cell apoptosis (Fig. 6B–I). Thus, TMEM176B function and PI3K-Akt-mTOR signaling dynamics are interdependent.

### In vivo* experiments confirmed that TMEM176B promotes GC*

**Fig. 7:**
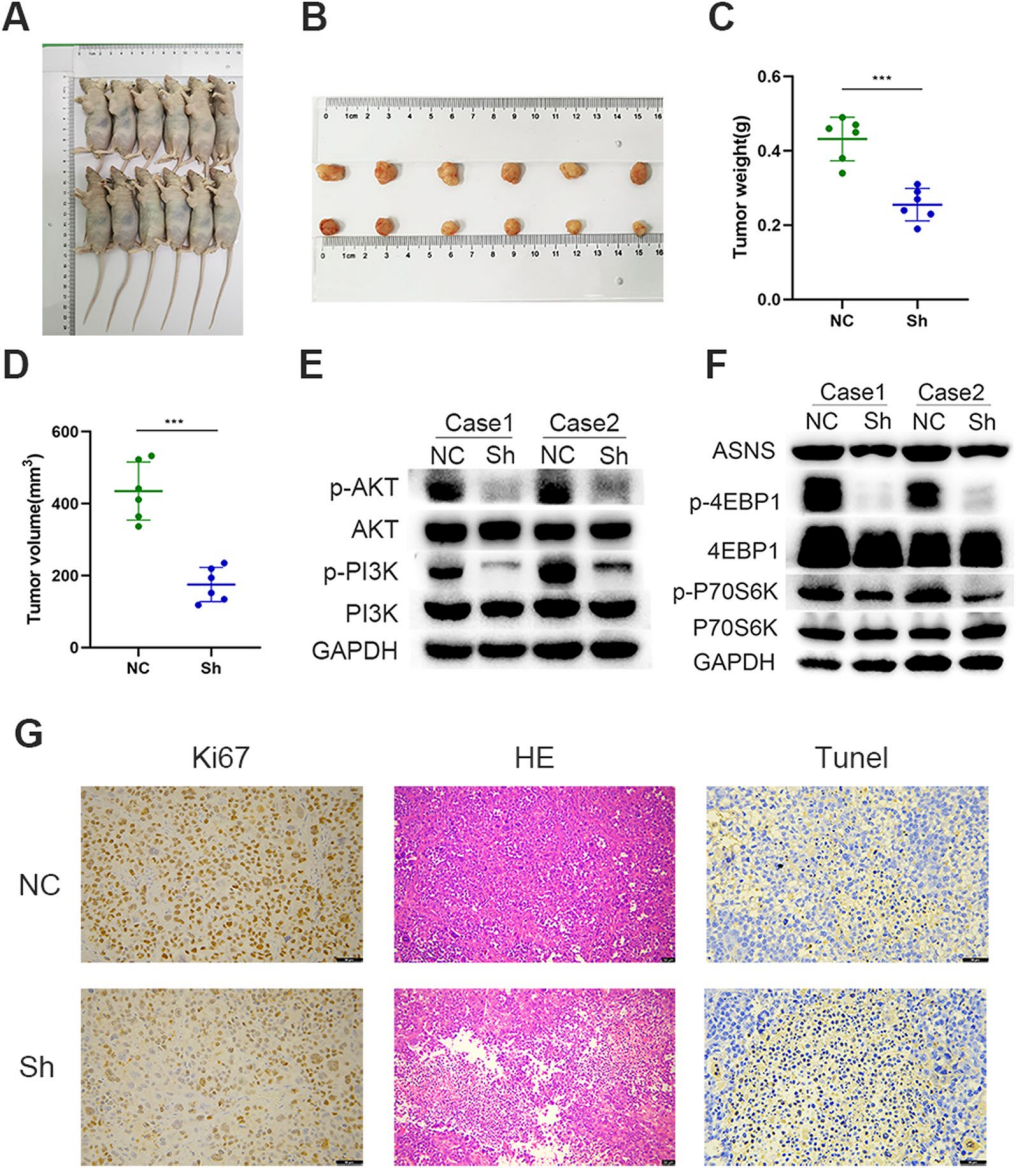
In vivo experiment corroborating the role of TMEM176B in GC progression. **A**, **B** Tumorigenesis was compared between the control and TMEM176B knockdown groups. **C**, **D** Differences in tumor volume and mass between control and TMEM176B knockdown groups were measured. **E**, **F** WB determined the expression levels of ASNS and proteins related to the PI3K-Akt-mTOR signaling pathway in the control and TMEM176B knockdown groups. **G** IHC and H&E staining were used to compare proliferation and apoptosis between the control and TMEM176B knockdown groups. ^*^p < 0.05, ^**^p < 0.01, ^***^p < 0.001

We conducted a xenograft tumor formation assay on athymic nude mice to validate the role of TMEM176B in GC progression. Six of the mice served as controls while another six were subjected to TMEM176B knockdown. There were substantially fewer neoplastic formations in the TMEM176B ablation cohort than in the control cohort (Fig. 7A, B). Moreover, tumor masses and volumes were smaller in the former than in the latter (Fig. 7C, D).

We then excised the mouse xenograft tumor tissues and found that the phosphorylation rates of the PI3K-Akt-mTOR signaling pathway constituents and the ASNS expression levels were markedly lower in the neoplasms of the TMEM176B knockdown mice than in those of the control mice (Fig. 7E, F).

IHC and hematoxylin–eosin (H&E) staining of the mouse xenograft tumors disclosed less GC cell proliferation and more GC cell apoptosis in the TMEM176B knockdown than in the control mice (Fig. 7G).

### TMEM176B upregulation in patients with GC indicated poor prognosis

**Fig. 8:**
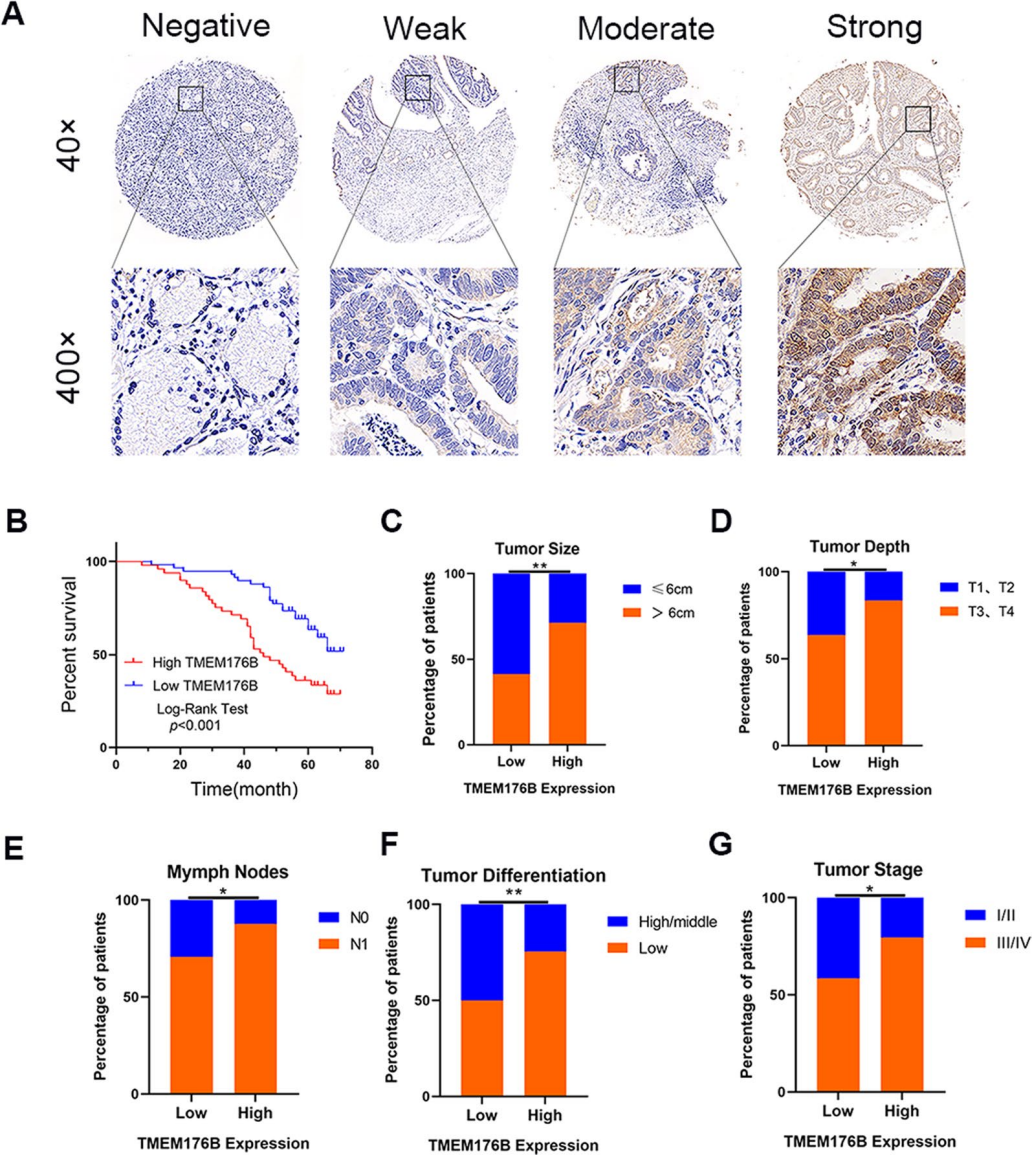
TMEM176B upregulation in patients with GC indicates poor prognosis. **A** Representative TMEM176B IHC staining images of GC tissue microarray (TMA). **B** Kaplan–Meier (K-M) survival curve of patients with GC and low- or high TMEM176B expression levels. **C**–**G** Correlations among TMEM176B expression and tumor size, depth, differentiation, and stage as well as lymph node metastasis. ^*^p < 0.05, ^**^p < 0.01, ^***^p < 0.001

We then used IHC to measure TMEM176B expression in a TMA comprising 107 GC and 22 proximate normal tissue samples (Fig. 8A). Based on the IHC staining indices, we categorized the patients with GC into cohorts with low and high TMEM176B expression levels.

We then correlated TMEM176B expression and the clinicopathological parameters in the GC cohort (Table 1). Survival analyses revealed a significant direct relationship between TMEM176B upregulation and OS reduction (p < 0.001; Fig. 8B). A χ^2^ test demonstrated significant positive correlations among TMEM176B expression and tumor size, GC cell invasion depth, lymph node metastasis, differentiation grade, and clinical staging (p < 0.05; Fig. 8C–G).

We then performed univariate and multivariate Cox regression analyses incorporating sex, age, tumor location and size, GC cell invasion depth, lymph node metastasis, differentiation grade, TNM staging, and TMEM176B expression. The univariate Cox regression analysis demonstrated significant correlations between overall survival (OS) and lymph node metastasis (p = 0.005), TNM staging (p = 0.039), and TMEM176B expression (p = 0.001) in the GC cohort (Table 2). The multivariate Cox regression analysis identified lymph node metastasis (p = 0.033) and TMEM176B expression (p = 0.009) as independent prognostic OS determinants in the GC cohort (Table 2).

**Table 2:** Univariate and multivariate analysis of clinicopathological variables and TMEM176B expression associated with overall survival

Parameters	Univariate analysis		Multivariate analysis	
	HR (95% CI)	*P*-value	HR (95% CI)	*P*-value
Gender (male vs female)	0.910(0.511–1.622)	0.750		
Age (years) (< 61 vs ≥ 61)	1.272 (0.732–2.212)	0.393		
Tumor location (upper vs middle + lower)	1.153 (0.643–2.068)	0.634		
Tumor size (cm) (< 6 vs ≥ 6)	0.824 (0.482–1.408)	0.479		
Depth of invasion (T1 + T2 vs T3 + T4)	1.578 (0.827–3.008)	0.166		
Lymph node metastasis (absent vs present)	3.713 (1.474–9.353)	0.005^a^	3.345 (1.103–10.145)	0.033^a^
Differentiation (well + moderate vs poor)	1.648 (0.918–2.957)	0.094		
TNM stage (I + II vs III + IV)	1.967 (1.034–3.739)	0.039^a^	0.844 (0.386–1.843)	0.670
TMEM176B expression (low vs high)	2.515 (1.452–4.357)	0.001^a^	2.124 (1.204–3.750)	0.009^a^

^a^Statistically significant (P < 0.05)

The preceding findings reflect the close association between TMEM176B expression and the clinicopathology landscape of patients with GC and suggest that TMEM176B overexpression indicates poor prognosis.

## Discussion

Advances have been made in reducing the incidence and mortality of gastric cancer (GC) in recent years. Nevertheless, there are few efficacious treatment options for this disease [41]. Hence, novel therapeutic targets are required for GC. Transmembrane protein 176B (TMEM176B) plays multiple complex roles in oncogenesis. Its overexpression drives breast cancer progression [23] while its knockdown enhances the efficacy of anticancer immune checkpoint inhibitors [22]. Moreover, its upregulation is paradoxically associated with improved melanoma prognosis. However, our in vitro and in vivo experiments revealed that TMEM176B significantly promotes GC progression. Patients with GC and low TMEM176B expression levels have relatively improved survival. Thus, TMEM176B is a potential therapeutic target for this disease.

Here, qRT-PCR and WB demonstrated that TMEM176B was overexpressed in GC cell lines and tissue samples (Fig. 1). TMEM176B knockdown inhibited proliferation, migration, and invasion (Fig. 2A) but promoted apoptosis (Fig. 2B–G and 3) in HGC27 and SGC7901 cell lines. Conversely, TMEM176B overexpression in AGS cell lines (Fig. 4A) had the opposite effects (Fig. 4B–I). Taken together, the foregoing data indicate that TMEM176B drives progression in GC cell lines.

We used bioinformatics analyses to clarify the mechanisms by which TMEM176B drives GC progression. Pathway enrichment analyses disclosed that the PI3K-Akt signaling pathway is strongly associated with TMEM176B (Fig. 5A, B). PI3K-Akt signaling plays crucial roles in a wide range of cancers [28, 42–45]. We used WB to evaluate the expression levels of PI3K-Akt signaling pathway proteins in GC cell lines subjected to TMEM176B knockdown and overexpression. TMEM176B knockdown and overexpression substantially decreased and increased PI3K-Akt phosphorylation, respectively (Fig. 5C). Downstream of the PI3K-Akt signaling pathway, mTOR regulates tumor growth, metabolism, immunity, and other processes [46]. P70S6K and 4EBP1 are essential components of mTOR [47]. Phosphorylation of the aforementioned mTOR proteins decreased and increased in response to TMEM176B knockdown and overexpression, respectively (Fig. 5D). In many cancers, mTOR is activated and controls cell growth and metabolism. The mTOR signaling pathway regulates AA, glucose, nucleotide, fatty acid (FA), and lipid metabolism [48]. An earlier study showed that mTOR is closely associated with ASNS [40]. We discovered that TMEM176B induces ASNS by inhibiting its ubiquitination degradation (Fig. 5D–F).

The addition of the mTOR inhibitor rapamycin in the presence of TMEM176B overexpression (Fig. 6A) partially weakened the potentiating effect of TMEM176B overexpression on GC cells (Fig. 6B–I). The preceding findings suggest that TMEM176B drives GC progression via the PI3K-Akt-mTOR signaling pathway. The results of our mouse tumor xenograft model supported this theory (Fig. 7).

Our survival and clinicopathological analyses established that TMEM176B expression is closely associated with the clinicopathology of patients with GC and confirmed that TMEM176B upregulation indicates poor prognosis (Fig. 8).

## Conclusion

Our in vitro, in vivo, and clinical experiments demonstrated that TMEM176B regulates ASNS metabolism through the PI3K-Akt-mTOR pathway and modulates GC progression. TMEM176B overexpression indicates the poor prognosis of GC and confirms that TMEM176B is a potential therapeutic target for this disease.

### Supplementary Information

**Additional file 1: Table S1.** Gene primer sequences.

## Data Availability

Not applicable.
