# Supplementary material for: Transmembrane protein 176B regulates amino acid metabolism through the PI3K-Akt-mTOR signaling pathway and promotes gastric cancer progression
Source: Cancer Cell Int. 2024 Mar 4;24:95. doi: 10.1186/s12935-024-03279-4 (PMC10913232; doi:10.1186/s12935-024-03279-4)
Supplement: Supplementary file 1 — Additional file 1: Table S1. Gene primer sequences. [file 12935_2024_3279_MOESM1_ESM.docx]

Supplementary Table 1 Gene primer sequences

| gene name | Primer sequence (5' -3 ') |
| --- | --- |
| TMEM176B | Forward: TGTCCTCTGCGTGAATAGCTTC |
|  | Reverse: CCATTGGTTCTCTTGACTTCGC |
| GAPDH | Forward: ATCAAGAAGGTGGTGAAGCAGG |
|  | Reverse: CGTCAAAGGTGGAGGAGTGG |
